# High-efficiency freezing-induced loading of inorganic nanoparticles and proteins into micron- and submicron-sized porous particles

**DOI:** 10.1038/s41598-018-35846-x

**Published:** 2018-12-10

**Authors:** Sergei V. German, Marina V. Novoselova, Daniil N. Bratashov, Polina A. Demina, Vsevolod S. Atkin, Denis V. Voronin, Boris N. Khlebtsov, Bogdan V. Parakhonskiy, Gleb B. Sukhorukov, Dmitry A. Gorin

**Affiliations:** 1 Skolkovo Institute of Science and Technology, Moscow, 143026 Russia; 2 Saratov State University, 83 Astrakhanskaya Str., Saratov, 410012 Russia; 3 Moscow Institute of Physics and Technology (State University), Dolgoprudny, Moscow Region 141701 Russia; 4 Shubnikov Institute of Crystallography of the Federal Scientific Research Centre “Crystallography and Photonics” of the Russian Academy of Sciences, Moscow, 119333 Russia; 5 Institute of Biochemistry and Physiology of Plants and Microorganisms, Saratov, 410049 Russia; 6 University of Ghent, 9000 Ghent, Belgium; 7 School of Engineering and Materials Science, Queen Mary University of London, London, E1 4NS UK

## Abstract

We demonstrate a novel approach to the controlled loading of inorganic nanoparticles and proteins into submicron- and micron-sized porous particles. The approach is based on freezing/thawing cycles, which lead to high loading densities. The process was tested for the inclusion of Au, magnetite nanoparticles, and bovine serum albumin in biocompatible vaterite carriers of micron and submicron sizes. The amounts of loaded nanoparticles or substances were adjusted by the number of freezing/thawing cycles. Our method afforded at least a three times higher loading of magnetite nanoparticles and a four times higher loading of protein for micron vaterite particles, in comparison with conventional methods such as adsorption and coprecipitation. The capsules loaded with magnetite nanoparticles by the freezing-induced loading method moved faster in a magnetic field gradient than did the capsules loaded by adsorption or coprecipitation. Our approach allows the preparation of multicomponent nanocomposite materials with designed properties such as remote control (e.g. via the application of an electromagnetic or acoustic field) and cargo unloading. Such materials could be used as multimodal contrast agents, drug delivery systems, and sensors.

## Introduction

Increasing the efficiency of loading of functional nanoparticles or molecular substances into porous particles is a currently central research problem. Solving this problem would permit investigators to improve carriers of bioactive substances for targeted delivery and to develop effective nanoparticle-based functional materials with superparamagnetic or plasmonic properties. There is a well-known method of pressing materials by the directed freezing of ice crystals, which is used to squeeze precursors for ceramic materials^1^. This method has not, to our knowledge, been used to load/press nanoparticles into a porous matrix of microparticles. In our work, we improved this method and tested it with a number of model nanoparticles commonly used to develop theranostic carriers with multifunctional properties. Such carriers can be navigated *in vivo* by magnetic field gradients^2,3^, and they provide the highest photoacoustic signal in undiluted blood^4^ and show laser activation^5^ and ultrasound-induced release^6^. The most widely used types of templates for such carriers are micron- and submicron-sized porous vaterite particles. These particles have significant advantages such as low cost, easily scaled-up preparation^7^, biocompatibility, biodegradability, solubility under soft pH conditions (6.5), high porosity^8^, and stability at high temperature and pressure^9^. Previous work has investigated the bioavailability and solubility of CaCO_3_ particles^10^ and their positive influence on the bioavailability of poorly water-soluble drugs^11^. The encapsulation of actual drugs instead of model fluorescent dyes or fluorescently labeled polymers has been reported. Examples of encapsulated substances include the anticancer agents cisplatin^12^ and doxorubicin^13^, tetracycline hydrochloride^14^, and photodynamic dyes^15^. Most published articles dealing with vaterite particles have reported *in vitro* studies of the uptake efficiency, toxicity (cell viability), and the kinetic release profiles of drugs^13,16,17^. Significant success in the efficiency of uptake and drug delivery is connected with transfer from micron to submicron range^18^.

Vaterite carriers began to be used *in vivo* more than eight years ago^19–21^. At present, they are used in biomedicine as templates and carriers for drug delivery systems. They are administered by various routes, including transdermal^19,22^, inhalation^20^, intertumoral^21^, and peroral^23^. Vaterite particles are also used in the preparation of food-friendly polymer additives^24^ and in water purification^25^. However, their transfer from R&D to industrial application has been made difficult by the lack of an efficient loading approach. Conventional methods allow only a maximum of 4 wt % of magnetite per seven steps, and the loading reproducibility for single steps is low. We demonstrate a method that provides a more efficient and controlled loading, as compared with other traditional approaches, such as adsorption from solution^26^, encapsulation during particle preparation (coprecipitation method)^16^, chemical vapor deposition^27^, and supercritical fluid technology^28^.

The freezing/thawing approach has been used widely for many purposes, including the fabrication of lamellar^29^ and porous structures, micro- and nanowires, and micro- and nanoparticles^1,30^. This approach is based on controlled directional freezing, so that ice crystals grow in a single direction^1^. In the a-direction of the hexagonal base, crystals grow 100 times faster than they grow in the perpendicular c-direction^31^. As a result, the ice crystals grow as a lamellar microstructure parallel to the a-direction, finally forming so-called cold-finger interfaces^31^.

Until now, this approach has not been used to load inorganic nanoparticles into micron and submicron porous particles. Here we propose the use of the cold finger approach for the freezing-induced loading (FIL) of inorganic nanoparticles into the porous matrices of vaterite particles.

## Discussion

**Figure 1:**
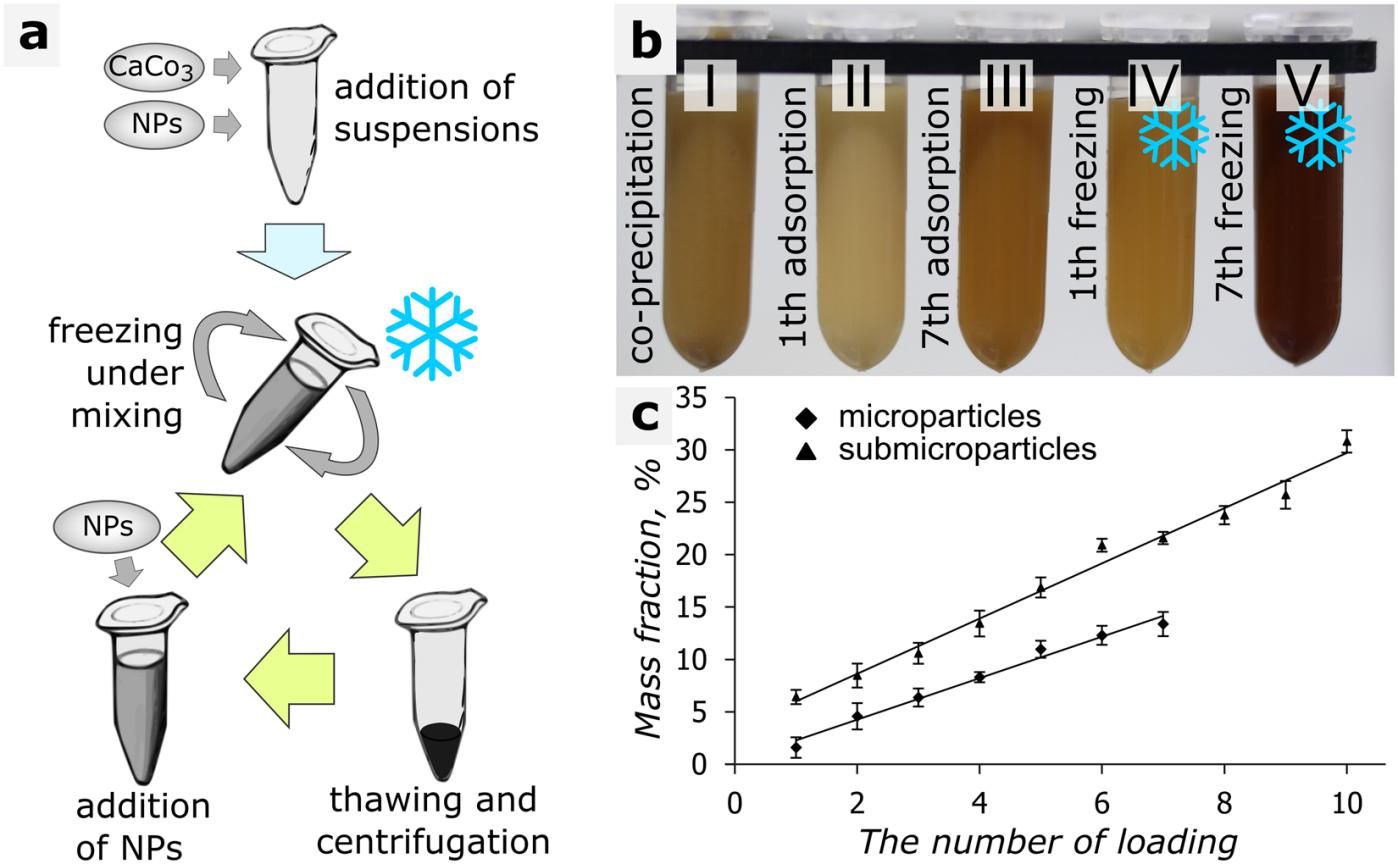
(**a**) Scheme of the FIL method; (**b**) (I), loading of MNPs by coprecipitation; (**b**) (II, III), 1st and 7th loading of MNPs by adsorption; (**b**) (IV, V), 1st and 7th loading of MNPs by FIL; (**c**), dependence of the MNP mass fraction (obtained by weighing the solid residue after each freezing-thawing cycle) on the number of freezing-thawing cycles.

In general, FIL includes several successive steps: addition of microparticle and nanoparticle suspensions to a polymeric centrifuge tube, freezing of samples under gentle mixing, thawing of samples, centrifugation of suspensions, and washing of suspensions with pure water. The process can be repeated several times, which makes it possible to obtain a high efficiency of nanoparticle loading (Fig. 1a).

This method allowed us to have up to 13 wt % of magnetite nanoparticles (MNPs) adsorbed into vaterite particles of micron and submicron sizes in a single freezing cycle. The vaterite microparticles, however, aggregated strongly in this case. Therefore, it was thought more practical to split the process into several freezing cycles, with smaller amounts of MNPs adsorbed during each cycle.

A comparative image of a vaterite suspension with MNPs loaded by different methods is shown in Fig. 1b. The image demonstrates the FIL method (Fig. 1b(IV,V)), as compared to the other methods used (Fig. 1b(I–III)). The FIL method allows higher amounts of magnetite (13%) adsorbed into vaterite microparticles, as compared to adsorption (4%) and coprecipitation (3%). After FIL, more nanoparticles of the same or different type can be added by the coprecipitation method^32^ or can be adsorbed into a polyelectrolyte shell^4,33,34^. This allows one to achieve an even higher loading or to construct multifunctional carriers.

Samples of vaterite particles were washed, dried, and weighed after each loading to estimate the mass fraction of the adsorbed MNPs. The dependence of the weight of the adsorbed MNPs on the number of freezing cycles is shown in Fig. 1c. The figure shows that if the mass ratios between MNPs and vaterite were the same, the quantity of adsorbed MNPs was greater on submicron-sized particles (mass fraction of 31%) than it was on micron-sized ones (mass fraction of 13%). Presumably, this is because submicron particles have a better developed porous surface^35,36^. At the same time, the total surface area of the micron-sized particles was eight times smaller than that of the submicron-sized particles.

**Figure 2:**
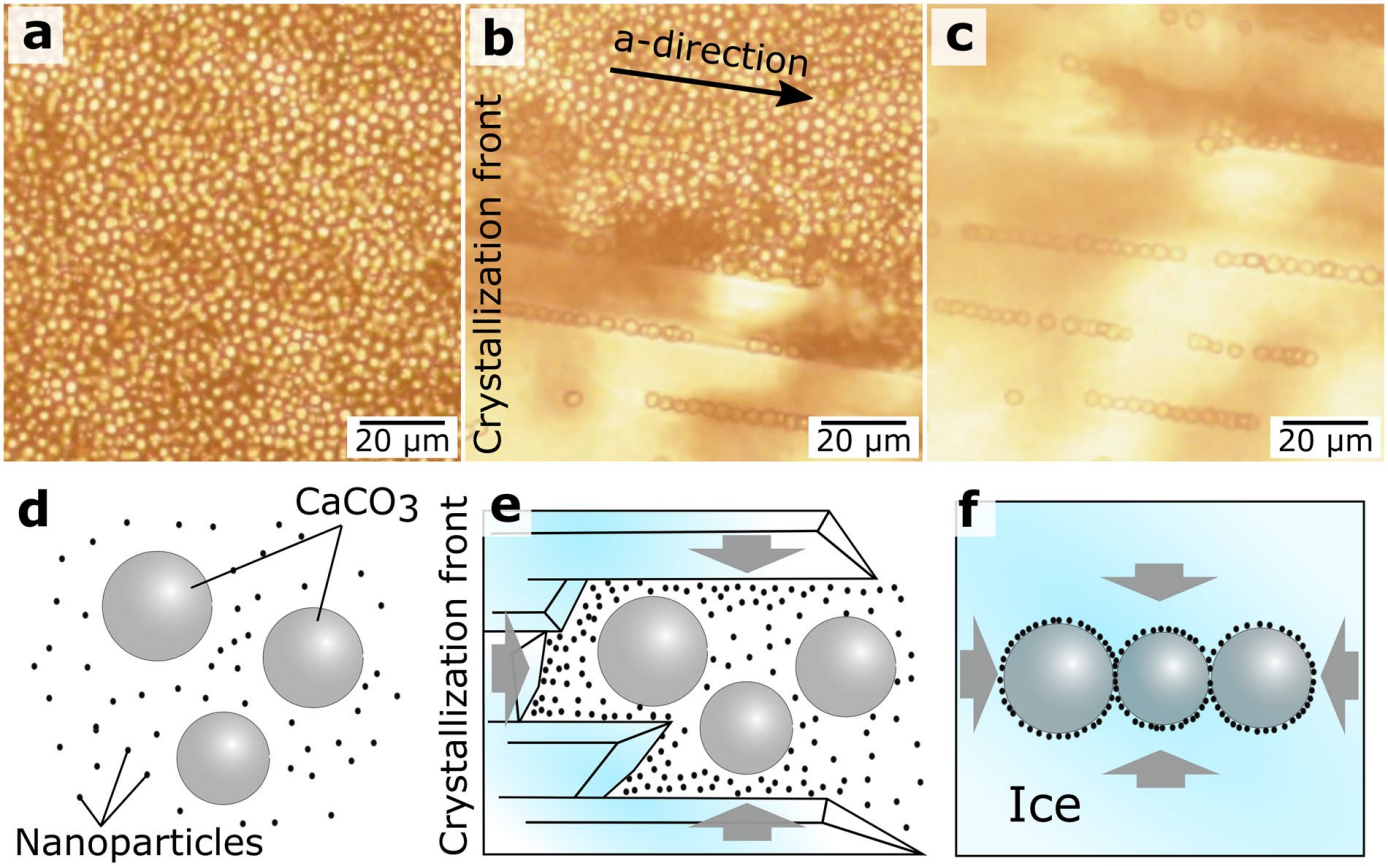
Optical microscopy of the FIL process. (**a**) Microparticle suspension; (**b**) Appearance of first ice fingers; (**c**) Pressing of nanoparticles into microparticles by ice fingers; (**d**–**f**) Scheme of MNP adsorption, induced by the movement of the crystallization front.

The freezing of a suspension containing CaCO_3_ microparticles and MNPs (see video in SI) is shown in Fig. 2. During crystallization, MNPs and CaCO_3_ microparticles were pushed by the crystallization front (Fig. 2b,e; ESI (video)), and the MNPs concentrated around the vaterite particles’ surface. In the final stage of the process (Fig. 2c,f; SI (video)), the MNPs were pressed, by the growing pressure of the forming ice, into the surface of the CaCO_3_ particles.

For pressing nanoparticles into the porous matrix of microparticles, both types of particles should be pushed forward by ice fingers and should remain untrapped by the crystallization front until all liquid phase is frozen. The particles are expelled by the crystallization front if the actual velocity of the front is less than the critical velocity at which particles are trapped during solidification^31^. The ratio of actual to critical freezing front velocities depends on the chemical composition of the crystallizing liquid and the particles, the particle radius, and the freezing conditions^31,37^. The suspensions of CaCO_3_ microparticles and MNPs were separately frozen to verify compliance with the above condition for each type of particle. Nano- and microparticles were moved off the tube walls by the crystallization front, which can easily be checked by visual observation (Supplementary Fig. S1, ESI (video)). This means that the actual velocity of the crystallization front under freezing of 2 mL of the suspension in a microcentrifuge tube placed into a freezing chamber at −20 °C was less than the critical freezing front velocity.

The presence of particles near the crystallization front leads to its distortion^3,31^. The solid–liquid interface undergoes convex bending toward a particle if that particle’s thermal conductivity is lower than that of the liquid. If the thermal conductivity of the particle is larger than that of the liquid, the solid–liquid interface undergoes concave bending away from the particle. In addition, regions are formed ahead of the crystallization front that have high concentrations of nano- and microparticles and a reduced crystallization point (concentration supercooling)^31,38^. The crystallization point of water in pores of less than 100 nm is lower than that in bulk water and decreases with decreasing pore diameter^39^. This effect can result in nanoparticles’ being pushed off into the vaterite pores by the crystallization front.

**Figure 3:**
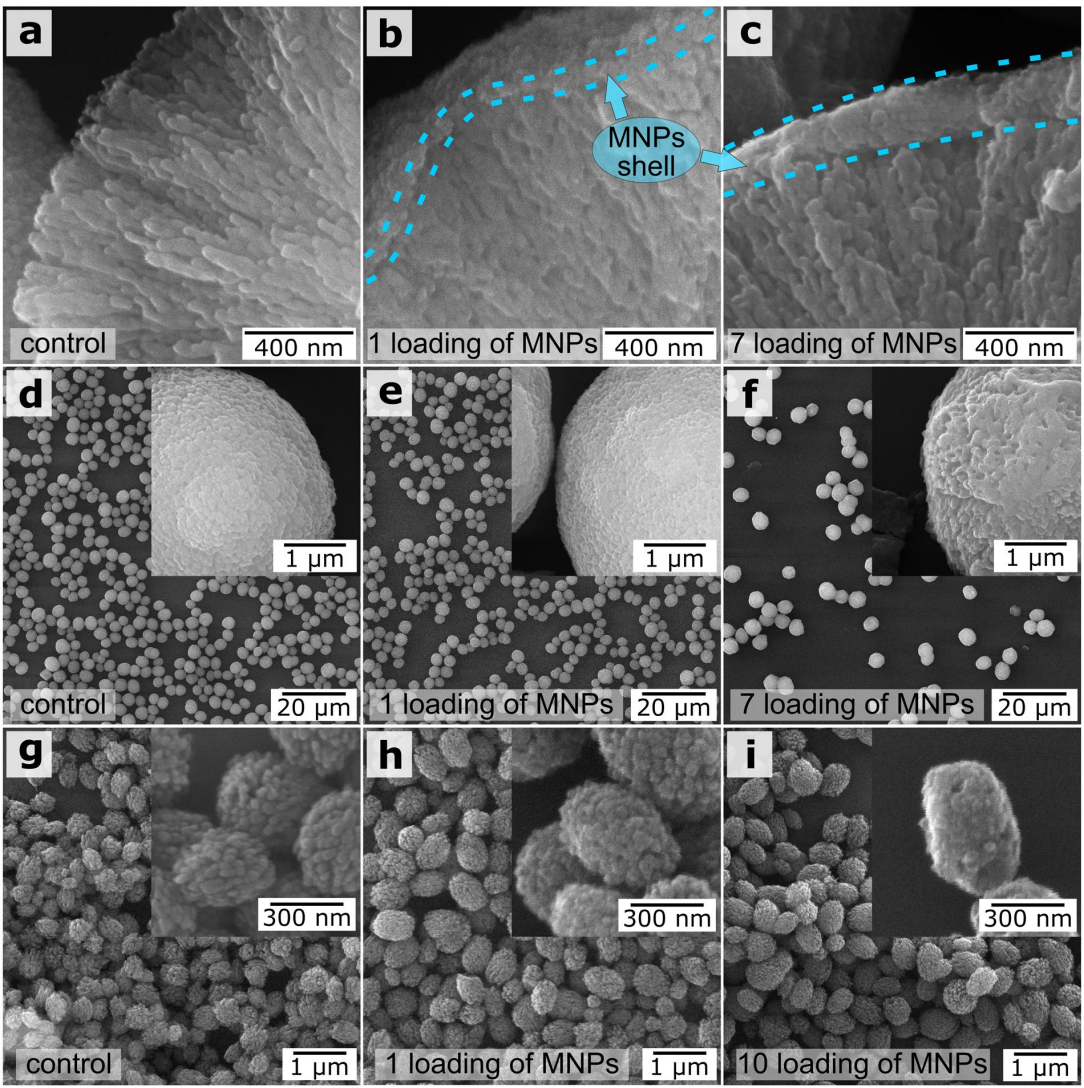
SEM images of micron (**a**–**f**) and submicron (**g**–**i**) vaterite particles. (**a**,**d**,**g**) Before FIL; (**b**,**e**,**h**) after one FIL cycle; (**c**,**f**,**i**) after the maximum number of FIL cycles. The insets show SEM images at higher magnification.

SEM images of vaterite microparticles before the FIL of MNPs (Fig. 3a) and after the 1st (Fig. 3b) and 7th (Fig. 3c) freezing/thawing cycles are shown in Fig. 3. The MNPs formed shells on the vaterite surface (Fig. 3b,c, blue dotted lines).

As shown in Fig. 3b,c,e,f,h,i, the shape of the micron and submicron vaterite particles was retained at each stage of the freezing/thawing cycle with MNPs. The calcite to vaterite ratio in the microparticles remained unchanged after each freezing/thawing cycle [confirmed by XRD measurements (Supplementary Fig. S3, Supplementary Table S1)]. These results are important for biomedical applications, because vaterite particles can dissolve under mild conditions and can become highly porous. As previously noted, vaterite particles are unstable and will recrystallize to calcite if they are not stabilized with polymeric shells^32^. Although the vaterite particles at the FIL stage did not have polymeric shells, they did not recrystallize, retaining their properties until shells were formed.

With the coprecipitation method, we noted a difference in the inclusion of MNPs into the pores of vaterite between submicron and micron particles^35,36^. As seen in Fig. 3d–i, the surface roughness of 3.5-µm vaterite particles was increased after each freezing-thawing cycle. For submicron particles, the surface roughness of the CaCO_3_ particles decreased with increasing number of freezing-thawing iterations owing to the gradual filling of the pores. This led to a smoothing of the particle surface. The total surface area of the micron-sized particles was eight times smaller than of the submicron ones weighing the same (Supplementary Equation S1). Therefore, the number of MNPs used in these experiments was insufficient to fill the pores and form continuous shells on the submicron particles.

**Figure 4:**
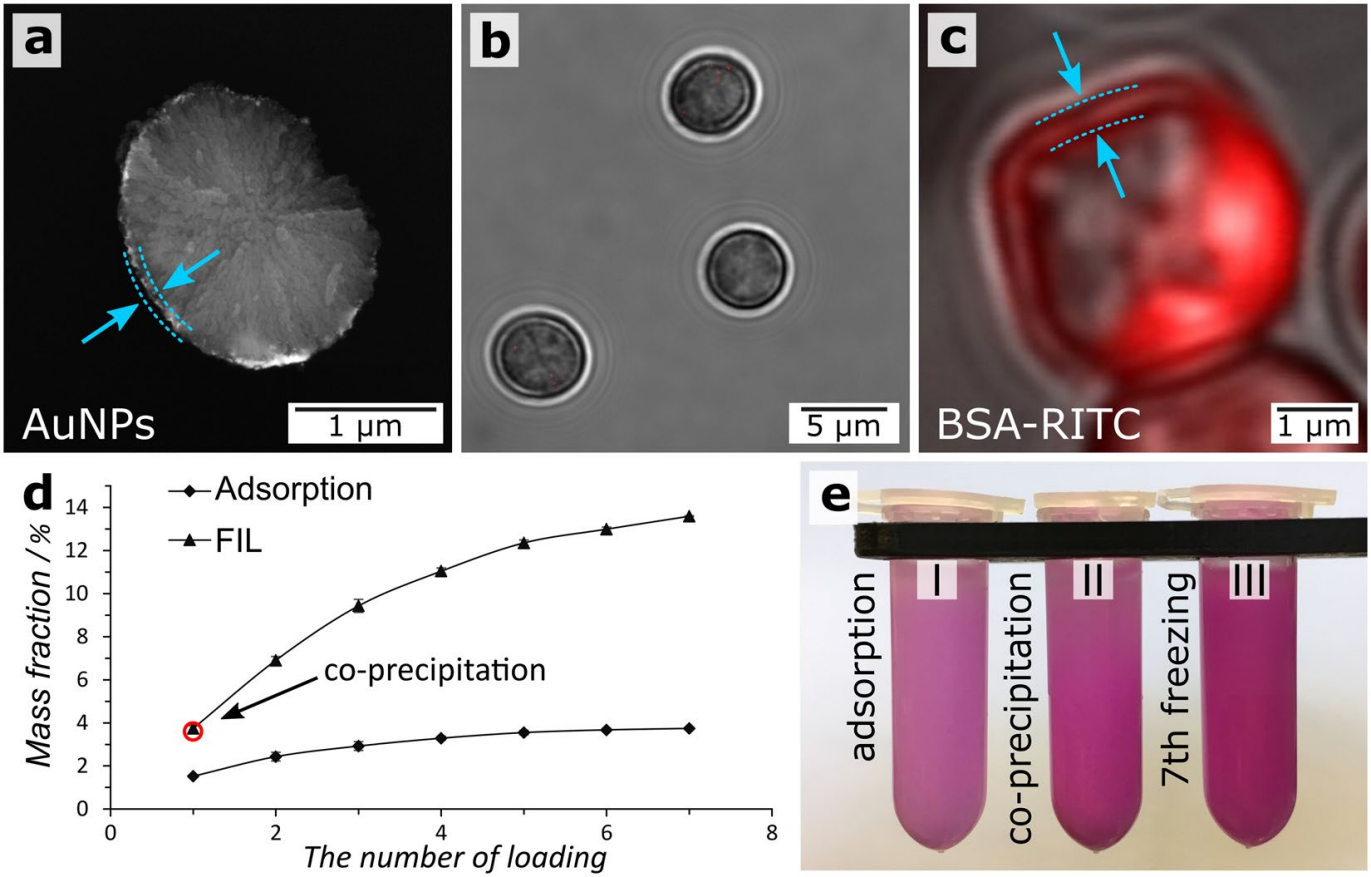
(**a**) SEM image of a microparticle loaded with AuNPs by FIL; (**b**) CLSM image of unloaded control microparticles; (**c**) CLSM image of a microparticle loaded with BSA–RITC by FIL; (**d**) dependence of the mass fraction of BSA–RITC on the number of freezing/thawing cycles or of adsorption steps. The arrow points to the mass fraction of BSA–RITC obtained by coprecipitation (empty red circle); (**e**) photo of the tubes containing CaCO_3_/BSA–RITC microparticles obtained by (I) adsorption, (II) coprecipitation, and (III) FIL.

The FIL method was tested on Au nanoparticles (AuNPs) to estimate the adsorption efficiency of particles of 10, 20, and 40 nm. The freezing/thawing loading process was repeated three times. The number of adsorbed particles was determined by UV–vis adsorption spectroscopy of the supernatant liquid after each freezing/thawing step. Adsorption was measured by the main AuNP adsorption line. It was found that the adsorption efficiency increased with increasing nanoparticle size (Supplementary Table S2). The AuNPs aggregated during loading, and the aggregates were placed on the surface of the microparticles (Fig. 4a; Supplementary Fig. S4). Supplementary Table S2 shows that the amount of AuNPs loaded by the FIL method was three times larger than that obtained through adsorption.

The FIL method can be used for the adsorption of polymers to the vaterite surface. It allows preparation of structures with a high amount of bovine serum albumin–rhodamine B isothiocyanate (BSA–RITC; Fig. 4e). BSA–RITC was adsorbed on microparticles from phosphate-buffered saline (PBS). The formation of BSA–RITC shells on the vaterite surface was confirmed by confocal laser scanning microscopy (CLSM; Fig. 4b,c), and the amount of adsorbed BSA–RITC was determined by spectrophotometry (the dependence of the mass fraction of BSA–RITC obtained by spectrophotometry on the number of FIL/adsorption loading cycles is shown in Fig. 4d). The FIL method allows adsorption of a four times larger amount of BSA–RITC (13.5%) than do the adsorption (3.7%) and coprecipitation (3.6%) methods. The freezing-thawing process can effect on the tertiary or quaternary structure of albumin. It can be controlled by factors such as the freezing rate and concentration of proteins. Increasing the freezing rate leads to a decrease in the time of strong influence of cold finger on the molecules^40^. In addition, growing the protein concentration enhances the protein stability. This can be explained by a decrease in the relative number of protein molecules at the ice/concentrated solution interface^41^. Thus, there are many possibilities for avoiding the negative influence of freezing on the tertiary or quaternary structure of albumin.

**Figure 5:**
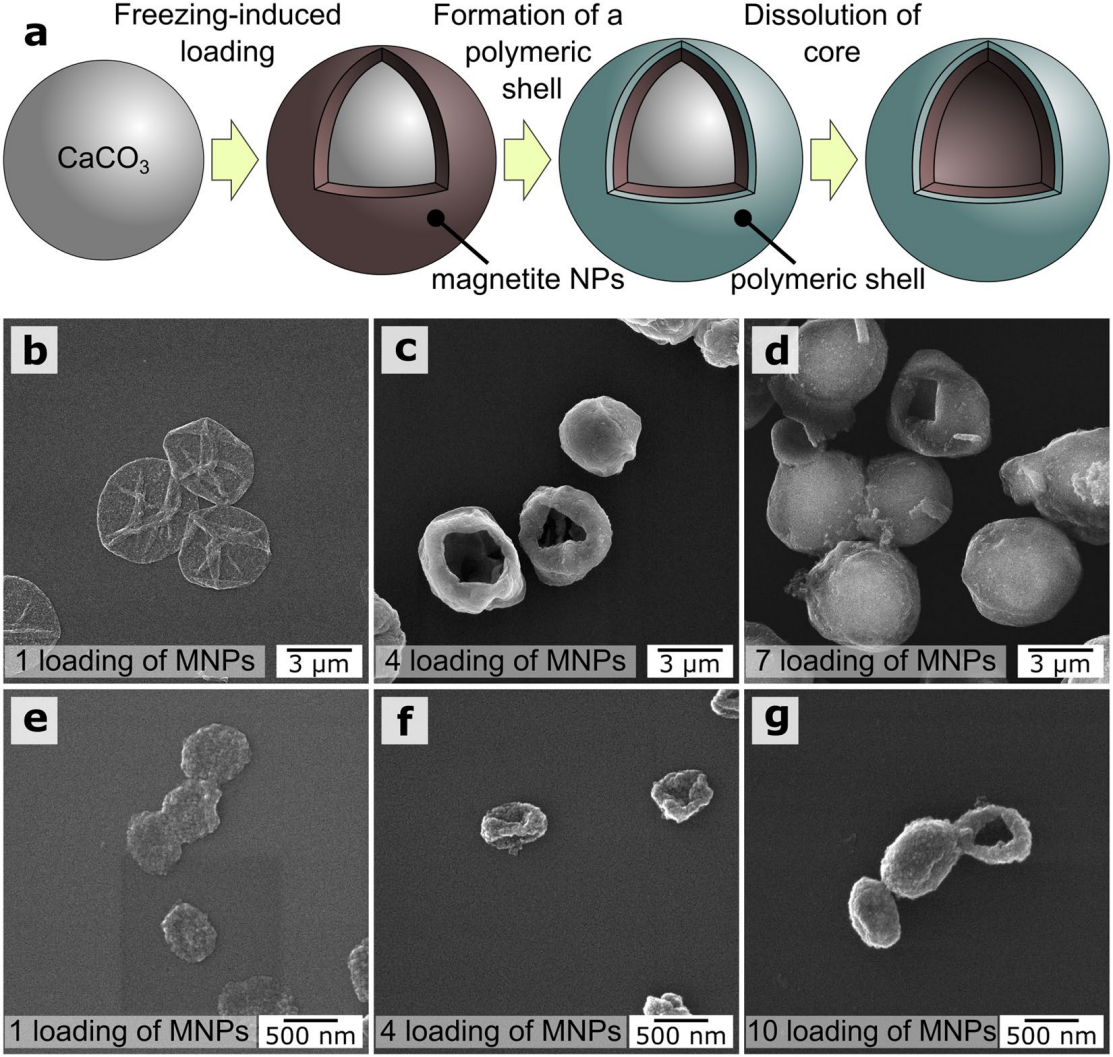
(**a**) Formation of polymeric capsules; (**b**–**d**) SEM images of microcapsules with different amounts of magnetite; (**e**–**g**) SEM images of submicron capsules with different amounts of magnetite.

The obtained micron- and submicron-sized composite particles were used as a template for polymeric magnetic capsules (Fig. 5a). The formation of polymeric capsules consisted of four stages. The first stage was the synthesis of micron or submicron vaterite particles. The second stage was the loading of different concentrations of MNPs on the vaterite particle surface by FIL. In the third stage, multilayered shells consisting of BSA and tannic acid were formed on the surface of the composite particles. These shells were formed by a complex of polyphenols and proteins interacting mostly through hydrogen bonds. They can be strong enough to be stable, although the electrostatic forces promoting interlayer interactions are not involved in the formation of these microspheres^42^. In the fourth stage, the vaterite cores were slowly dissolved by adding 0.2 M EDTA solution; this was followed by a washing step. This procedure resulted in micron- and submicron-sized polymeric capsules with different amounts of magnetite (Fig. 5b–g).

Figure 5d,g shows that the capsules formed on the basis of vaterite microparticles loaded with a large amount of magnetite did not collapse after drying. This indirectly indicates that the bulk of the magnetite particles was distributed over the vaterite particle surface and formed magnetite shells/crusts, which retained their shape after the core dissolved. Compared to the micron-sized capsules, the submicron capsules did not retain their shape on drying.

**Figure 6:**
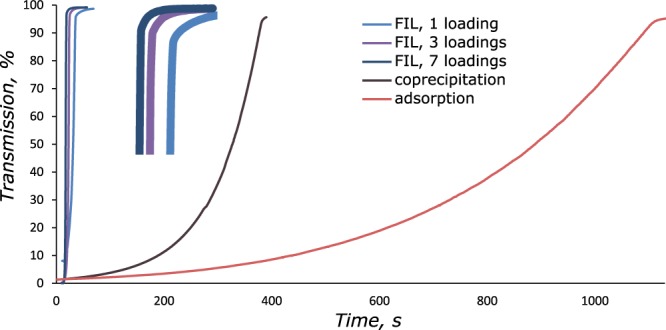
Time dependence of the extinction of the microcapsule solution in an external magnetic field.

The movement rate of the obtained capsules in an external magnetic field was measured as the time dependence of sample extinction at 660 nm. The dependences are plotted in Fig. 6. The measurements were carried out with a setup presented in Supplementary Fig. S5. As shown in Fig. 6, FIL allows preparation of polymeric nanocomposite capsules with large amounts of magnetite and, as a result, with a high magnetic moment for better magnetic navigation in drug delivery applications.

The release of BSA-RITC from the obtained microcapsules was measured for 9 days. The time dependence of the amount of released BSA-RITC is shown in Supplementary Fig. S6. As can be seen, the release of BSA-RITC was slow and sustained. However, the permeability of polymeric shells depends on their thickness, polymer molecular weight, pH, and ionic strength of the solution^43^. The loaded material can also be released in a controlled manner by enzymatic shell degradation^34,42^, laser radiation^5^, or ultrasound treatment^6^.

## Conclusions

The method offered in this article shows good promise for R&D and the fabrication of new, innovative products for the pharma, aerospace, photonics, environment, and food industries. Progress in this direction is hindered by the lack of methods for the preparation of multifunctional smart materials. Multifunctionality of materials and control of their properties can be implemented by using multicomponent composite structures with precisely defined mass fractions and distribution of every component. The FIL method permits adsorption of components in a quantitive manner with high reproducibility for every cycle, as demonstrated here with AuNPs, MNPs, and BSA–RITC. In addition, the number of loaded MNPs increases with decreasing vaterite particle size. The FIL method affords at least a three times higher loading of magnetite nanoparticles and a four times higher loading of protein into micron-sized vaterite particles, in comparison with conventional methods such as adsorption and coprecipitation. For submicron particles, FIL method allows five times higher loading of magnetite nanoparticles in comparison with conventional methods.

Capsules loaded with MNPs by FIL were fabricated. These capsules moved faster in a magnetic field gradient than did capsules loaded by adsorption or coprecipitation.

In summary, this cheap and easy method can be used to make composite fillers for new alloys, multimodal contrast agents, sensors, and delivery carriers of bioactive substances with tunable sensitivities to external stimuli (magnetic field gradients, laser irradiation, or ultrasound). Such carriers could include polymeric capsules^42,44^ or oxygen carriers^45^ with much-increased mass fractions of active substances.

## Methods

Iron(III) chloride hexahydrate (99.8%), iron(II) chloride tetrahydrate (99.8%), citric acid (99.8%), BSA, tannic acid, hydrochloric acid, calcium chloride dehydrate, anhydrous sodium carbonate, and sodium chloride were purchased from Sigma-Aldrich. Sodium hydroxide (99.8%) and disodium ethylenediaminetetraacetate dihydrate (EDTA) were purchased from Fluka. All chemicals were used without further purification. Deionized (DI) water (specific resistivity higher than 18.2 MΩ∙cm) from a Millipore Milli-Q Direct 8 system was used to make all solutions.

### Preparation of magnetite nanoparticles

MNPs were prepared by chemical precipitation from a mixed solution of bi- and trivalent iron salts in basic media, as described by Massart^46^. An automatic chemical reactor was used to conduct the chemical reaction and the subsequent stabilization of the nanoparticles^47^. The MNPs had a size of 11 ± 5 nm and a zeta potential of −29 ± 5 mV. The nanocomposite structures containing this type of magnetite nanoparticles have saturation magnetization up to M_s_ = 72 emu/g and exhibit the superparamagnetic behavior^48^. The magnetite concentration was 1.25 mg/mL, as measured by the solid residue method.

### Preparation of vaterite particles

Spherical porous CaCO_3_ microparticles were synthesized according to Volodkin *et al*.^49^. In brief, 0.615 mL of 1 M CaCl_2_ and 0.615 mL of 1 M Na_2_CO_3_ solutions were injected into 2.5 mL of DI water under vigorous agitation. One min later the agitation was stopped, and the resulting dispersion of particles was separated by centrifugation and washed two times with DI water.

Submicron particles were prepared in 4 mL of glycerol, after which 400 µL of 0.5 M CaCl_2_ and 400 µL of 0.5 M Na_2_CO_3_ solutions were added with constant stirring (500 rpm).

The size of the CaCO_3_ microparticles was 3.6 ± 0.5 µm, and the submicron particles were prolate spheroids with a size of a = 350 ± 40 nm and b = 230 ± 40 nm. As mentioned before, vaterite particles are unstable and will recrystallize if stored in water. To prevent recrystallization, we dehydrated the samples with acetone and dried them afterwards. It is more practical to store vaterite particles dried and resuspend them before use.

### Loading of vaterite particles

CaCO_3_ microparticles were loaded by coprecipitation as follows: 0.615 mL of 1 M CaCl_2_ and 0.615 mL of 1 M Na_2_CO_3_ solutions were injected into 2.5 mL of an MNP suspension under vigorous agitation. One min later the agitation was stopped, and the resulting dispersion of particles was separated by centrifugation and washed two times with DI water.

CaCO_3_ microparticles were loaded by adsorption as follows: 2 mL of an MNP suspension was added to 40 mg of CaCO_3_ particles, and the mixture was shaken for 15 min. After that, the particles were separated by centrifugation and washed two times with DI water. The procedure was repeated up to seven times for different samples.

CaCO_3_ microparticles were loaded by FIL as follows: 2 mL of an MNP suspension was added to 40 mg of CaCO_3_ particles. A microcentrifuge tube with the reaction mixture was kept in a freezing chamber at −20 °C for 2 h and was stirred slowly and constantly. After that, the samples were thawed at room temperature and washed. Some of the samples were dried in a drying cabinet, while others were subjected to repeated freezing/thawing. Freezing/thawing was repeated up to seven times for different samples.

The amount of MNPs loaded by FIL was measured as the difference between the weights of the particles before and after loading. In coprecipitation, the MNP amount was determined by colorimetric titration, because in this case the MNPs were loaded during the preparation of vaterite microparticles. The loading efficiency of the AuNPs was measured by UV–vis spectroscopy (Synergy H1Multi-Mode Reader, BioTek Instruments, USA) by measuring light adsorption in the 300–900 nm range. After each freezing/thawing step, we measured the amount of unloaded AuNPs in the supernatant liquid. The amount of loaded AuNPs was determined as the difference between the initial and unloaded amounts of the particles. The BSA–RITC concentration was determined by measuring the fluorescence intensity (*λ*_ex_ = 560 nm, *λ*_em_ = 590 nm) of the supernatant liquid after each freezing/thawing step. All measurements were made at 24 °C in 96-well disposable plates.

Washing of a suspension after thawing and centrifugation leads to the loss of a certain number of vaterite particles. For correction of the obtained mass fraction values, we determined the weight of particles lost at each step in a control experiment, by using submicron and micron vaterite particles without MNPs.

### Preparation of polymeric capsules

The preparation of composite shells is a multistage process. The template for polymeric capsules were vaterite particles loaded with MNPs. Layer-by-layer assembly was used to make micron and submicron capsules^35,36,44^. Microcapsules were prepared by sequential adsorption of 1 mL of BSA (concentration of 2 mg/mL in water) and tannic acid (concentration of 2 mg/mL in water) onto the spherical surfaces of CaCO_3_ cores. Every polymer adsorption cycle was conducted for 15 min. The cores were then gently dissolved by treatment with EDTA (concentration of 0.2 M in water, pH 7.3), and the residues were removed by two times washing in DI water. For gentle core dissolution, EDTA was slowly added to the particle suspension under shaking until the core disappeared. After each adsorption step, as well as after the dissolution of the CaCO_3_ cores, the suspension of the microparticles was centrifuged (at 240 g for micron capsules and at 1300 g for submicron capsules) and was washed twice with pure water^42^. As a result, the capsules had shells with three bilayers of BSA–tannic acid. In contrast to vaterite particles, the polymeric composite capsules are very stable. All samples of composite capsules were kept in a freezer at 4 °C. After 15 months of storage, the capsules retained their shape and zeta potential (−45 ± 5 mV) and did not aggregate.

### Scanning electron microscopy

Scanning electron microscopy (SEM) analysis was done with a MIRA II LMU instrument (Tescan, Brno, Czech Republic). Samples were prepared by depositing a drop of a particle or capsule suspension on a silicon wafer and leaving it to dry at room temperature. Before imaging, the samples were coated with an about 5-nm-thick Au film by using an Emitech K350 sputter coater. Images were taken at 30 kV.

### Optical microscopy

Confocal laser scanning microscopy (CLSM) was done with a Leica TCS SP8 X instrument (Leica, Germany) equipped with a x100/1.44 oil immersion objective. A laser wavelength of 552 nm was used to excite BSA–RITC fluorescence.

The expulsion of microparticles by the crystallization front was visualized with an Olympus IX73 optical inverted microscope and with a Leica DM2500 upright microscope. Both microscopes were operated in the bright-field mode.

### Raman measurements

These were made with dried samples on fused silica, by using a Renishaw inVia spectrometer with a 50x/0.5 N.A. long working distance objective lens. The laser excitation was at 532 nm, the power was 0.3 mW, and the detection time was 60 s.

### X-ray diffraction measurements

The samples were studied by X-ray diffraction on an X’pert Pro (МPD) diffractometer (PANanalytical, Netherlands) (Ge monochromator) in CuK_α_ radiation (0.154 nm) in the continuous mode. A qualitative diffraction analysis of the samples was done by using the JCPDS PDF-2 database and original studies.

## Electronic supplementary material

Supplementary information

ESI video

## References

[1] Qian L, Zhang H (2011). Controlled freezing and freeze drying: a versatile route for porous and micro-/nano-structured materials. J. Chem. Technol. Biotechnol..

[2] Voronin DV (2017). *In Vitro* and *in Vivo* Visualization and Trapping of Fluorescent Magnetic Microcapsules in a Bloodstream. ACS Appl. Mater. Interfaces.

[3] Galanzha EI (2012). *In vivo* magnetic enrichment, photoacoustic diagnosis, and photothermal purging of infected blood using multifunctional gold and magnetic nanoparticles. PLoS One.

[4] Yashchenok AM, Jose J, Trochet P, Sukhorukov GB, Gorin DA (2016). Multifunctional polyelectrolyte microcapsules as a contrast agent for photoacoustic imaging in blood. J. Biophotonics.

[5] Gorin DA (2008). Magnetic/gold nanoparticle functionalized biocompatible microcapsules with sensitivity to laser irradiation. Phys. Chem. Chem. Phys..

[6] Shchukin DG, Gorin DA, Möhwald H (2006). Ultrasonically induced opening of polyelectrolyte microcontainers. Langmuir.

[7] Svenskaya YI (2016). Ultrasonically assisted fabrication of vaterite submicron-sized carriers. Adv. Powder Technol..

[8] Trushina DB, Bukreeva TV, Kovalchuk MV, Antipina MN (2014). CaCO3 vaterite microparticles for biomedical and personal care applications. Mater. Sci. Eng. C.

[9] Maciejewski M, Oswald H-R, Reller A (1994). Thermal transformations of vaterite and calcite. Thermochim. Acta.

[10] Meiron OE (2011). Solubility and bioavailability of stabilized amorphous calcium carbonate. J. Bone Miner. Res..

[11] Forsgren J, Andersson M, Nilsson P, Mihranyan A (2013). Mesoporous calcium carbonate as a phase stabilizer of amorphous celecoxib–an approach to increase the bioavailability of poorly soluble pharmaceutical substances. Adv. Healthc. Mater..

[12] Dunuweera SP, Rajapakse RMG (2017). Encapsulation of anticancer drug cisplatin in vaterite polymorph of calcium carbonate nanoparticles for targeted delivery and slow release. Biomed. Phys. Eng. Express.

[13] Bosio VE (2014). Synthesis and characterization of CaCO3–biopolymer hybrid nanoporous microparticles for controlled release of doxorubicin. Colloids Surfaces B Biointerfaces.

[14] Mihai M (2017). Autotemplate Microcapsules of CaCO3/Pectin and Nonstoichiometric Complexes as Sustained Tetracycline Hydrochloride Delivery Carriers. ACS Appl. Mater. Interfaces.

[15] Svenskaya Y (2013). Anticancer drug delivery system based on calcium carbonate particles loaded with a photosensitizer. Biophys. Chem..

[16] Svenskaya YI (2016). Photodynamic therapy platform based on localized delivery of photosensitizer by vaterite submicron particles. Colloids Surfaces B Biointerfaces.

[17] Dunuweera SP, Rajapakse RMG (2017). Synthesis of Unstable Vaterite Polymorph of Porous Calcium Carbonate Nanoparticles, Encapsulation of Anticancer Drug Cisplatin, Studying Release Kinetics for Safe, Targeted Delivery and Slow Release. *J Nanomedine Biother*. Discov.

[18] Parakhonskiy BV, Haase A, Antolini R (2012). Sub-Micrometer Vaterite Containers: Synthesis, Substance Loading, and Release. Angew. Chemie Int. Ed..

[19] Genina EA (2016). *In vivo* optical monitoring of transcutaneous delivery of calcium carbonate microcontainers. Biomed. Opt. Express.

[20] Gusliakova, O. *et al*. Use of submicron vaterite particles serves as an effective delivery vehicle to the respiratory portion of the lung. *Front. Pharmacol*. **9** (2018). 10.3389/fphar.2018.00559 PMC5994594 29915536

[21] Svenskaya YI (2014). Calcium carbonate microparticles containing a photosensitizer Photosens: preparation, ultrasound stimulated dye release, and *in vivo* application. Nanotechnologies Russ..

[22] Liu D (2018). Fabrication of composite microneedles integrated with insulin-loaded CaCO3 microparticles and PVP for transdermal delivery in diabetic rats. Mater. Sci. Eng. C.

[23] Kilic E (2017). Formulation for oral delivery of lactoferrin based on bovine serum albumin and tannic acid multilayer microcapsules. Sci. Rep..

[24] Abebe M, Hedin N, Bacsik Z (2015). Spherical and porous particles of calcium carbonate synthesized with food friendly polymer additives. Cryst. Growth Des..

[25] Nakamura J, Kasuga T, Sakka Y (2017). Preparation of carbamate-containing vaterite particles for strontium removal in wastewater treatment. J. Asian Ceram. Soc..

[26] De Cock LJ (2010). Polymeric multilayer capsules in drug delivery. Angew. Chemie Int. Ed..

[27] Christian P, Ehmann HMA, Coclite AM, Werzer O (2016). Polymer Encapsulation of an Amorphous Pharmaceutical by initiated Chemical Vapor Deposition for Enhanced Stability. ACS Appl. Mater. Interfaces.

[28] Liu H, Finn N, Yates MZ (2005). Encapsulation and sustained release of a model drug, indomethacin, using CO2-based microencapsulation. Langmuir.

[29] Han J, Hu L, Zhang Y, Zhou Y (2009). Fabrication of Ceramics with Complex Porous Structures by the Impregnate–Freeze-Casting Process. J. Am. Ceram. Soc..

[30] Zhang H, Cooper AI (2007). Aligned porous structures by directional freezing. Adv. Mater..

[31] Wegst UGK, Schecter M, Donius AE, Hunger PM (2010). Biomaterials by freeze casting. Philos. Trans. R. Soc. London A Math. Phys. Eng. Sci..

[32] Sergeeva A (2015). Composite magnetite and protein containing CaCO3 crystals. External manipulation and vaterite> calcite recrystallization-mediated release performance. ACS Appl. Mater. Interfaces.

[33] Andreeva DV, Gorin DA, Shchukin DG, Sukhorukov GB (2006). Magnetic microcapsules with low permeable polypyrrole skin layer. Macromol. Rapid Commun..

[34] German SV (2016). *In vitro* and *in vivo* MRI visualization of nanocomposite biodegradable microcapsules with tunable contrast. Phys. Chem. Chem. Phys..

[35] Trushina DB, Bukreeva TV, Antipina MN (2016). Size-controlled synthesis of vaterite calcium carbonate by the mixing method: aiming for nanosized particles. Cryst. Growth Des..

[36] Sukhorukov GB (2004). Porous calcium carbonate microparticles as templates for encapsulation of bioactive compounds. J. Mater. Chem..

[37] Scherer GW (1999). Crystallization in pores. Cem. Concr. Res..

[38] Zhang H (2005). Aligned two-and three-dimensional structures by directional freezing of polymers and nanoparticles. Nat. Mater..

[39] Ashworth EN, Abeles FB (1984). Freezing behavior of water in small pores and the possible role in the freezing of plant tissues. Plant Physiol..

[40] Yu Z, Garcia AS, Johnston KP, Williams RO (2004). Spray freezing into liquid nitrogen for highly stable protein nanostructured microparticles. Eur. J. Pharm. Biopharm..

[41] Tang XC, Pikal MJ (2005). The effect of stabilizers and denaturants on the cold denaturation temperatures of proteins and implications for freeze-drying. Pharm. Res..

[42] Lomova MV (2015). Multilayer capsules of bovine serum albumin and tannic acid for controlled release by enzymatic degradation. ACS Appl. Mater. Interfaces.

[43] Antipov AA, Sukhorukov GB (2004). Polyelectrolyte multilayer capsules as vehicles with tunable permeability. Adv. Colloid Interface Sci..

[44] Sukhorukov GB (1998). Stepwise polyelectrolyte assembly on particle surfaces: a novel approach to colloid design. Polym. Adv. Technol..

[45] Xiong Y (2013). Nonvasoconstrictive hemoglobin particles as oxygen carriers. ACS Nano.

[46] Massart R (1981). Preparation of aqueous magnetic liquids in alkaline and acidic media. IEEE Trans. Magn..

[47] German SV (2013). Synthesis of magnetite hydrosols in inert atmosphere. Colloid J..

[48] Dincer I (2012). Effect of the number of iron oxide nanoparticle layers on the magnetic properties of nanocomposite LbL assemblies. J. Magn. Magn. Mater..

[49] Volodkin DV, Larionova NI, Sukhorukov GB (2004). Protein encapsulation via porous CaCO3 microparticles templating. Biomacromolecules.

