# Supplementary material for: High-efficiency freezing-induced loading of inorganic nanoparticles and proteins into micron- and submicron-sized porous particles
Source: Sci Rep. 2018 Dec 10;8:17763. doi: 10.1038/s41598-018-35846-x (PMC6288109; doi:10.1038/s41598-018-35846-x)
Supplement: Supplementary file 1 — Supplementary information [file 41598_2018_35846_MOESM1_ESM.pdf]

# Supplementary information

## High-efficiency freezing-induced loading of inorganic nanoparticles and proteins into micron- and submicron-sized porous particles

Sergei V. German,[1,2] Marina V. Novoselova,[1,2] Daniil N. Bratashov,[2,3] Polina A. Demina,[2,4] Vsevolod S. Atkin,[2] Denis V. Voronin[2], Boris N. Khlebtsov,[5,2] Bogdan V. Parakhonskiy,[6,2] Gleb B. Sukhorukov,[7,2] and Dmitry A. Gorin*[1,2]

1 Skolkovo Institute of Science and Technology, Moscow, 143026, Russia

2 Saratov State University, 83 Astrakhanskaya Str., Saratov, 410012, Russia

3 Moscow Institute of Physics and Technology (State University), Dolgoprudny, Moscow Region, 141701, Russia

4 Shubnikov Institute of Crystallography of the Federal Scientific Research Centre "Crystallography and Photonics" of the Russian Academy of Sciences, Moscow, 119333, Russia

5 Institute of Biochemistry and Physiology of Plants and Microorganisms, Saratov, 410049, Russia

6 University of Ghent, 9000 Ghent, Belgium

7 School of Engineering and Materials Science, Queen Mary University of London, London, E1 4NS, UK

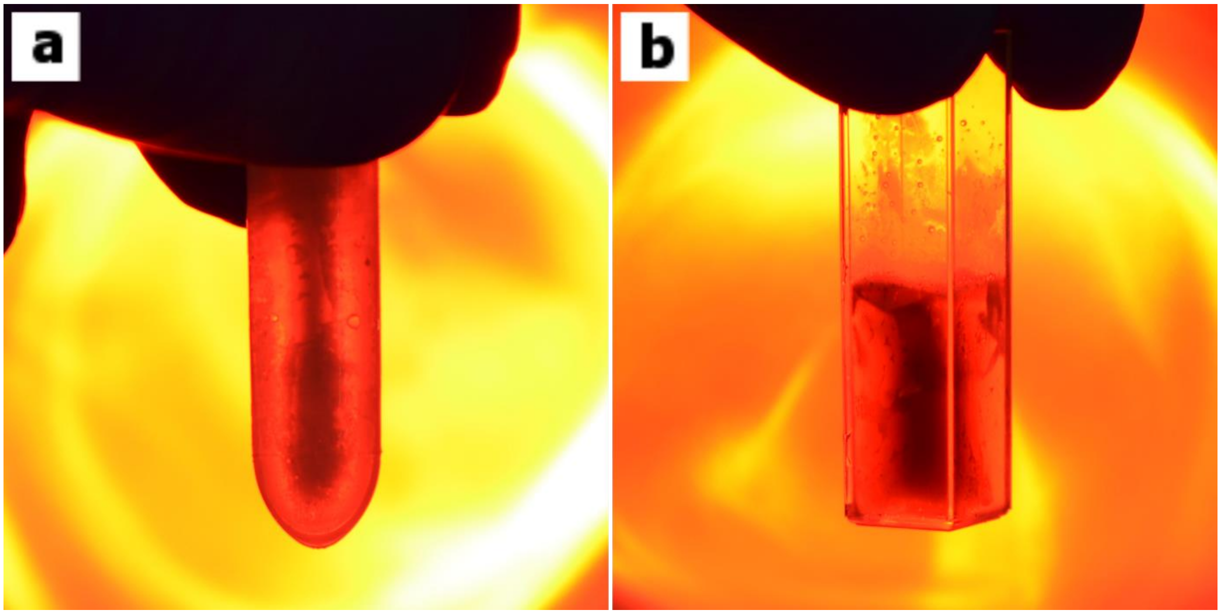


**Supplementary Figure S1.** Separately frozen suspensions of calcium carbonate microparticles (a) and magnetite nanoparticles (b) with the same conditions. Nano- and microparticles were moved off tube walls by crystallization front.

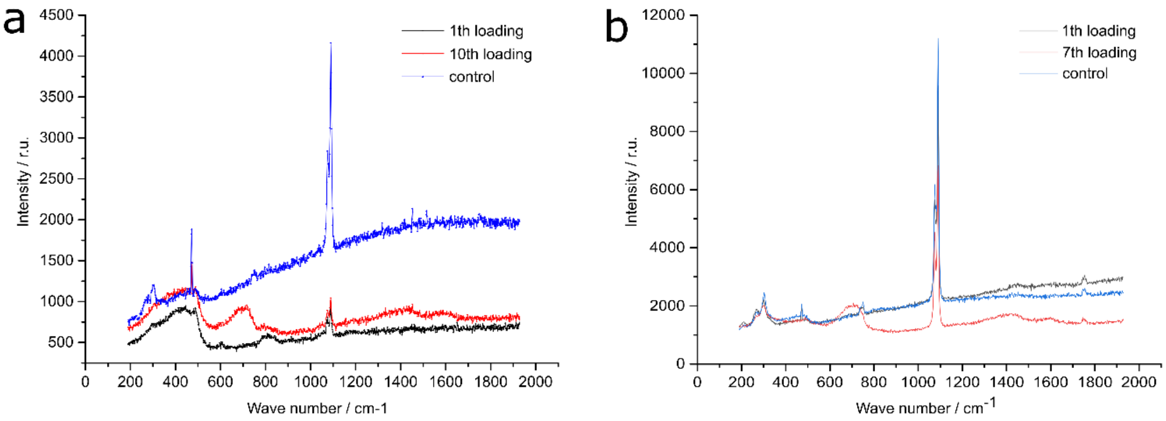


**Supplementary Figure S2.** Raman spectra of vaterite microparticles without magnetite (control – blue lines) and with magnetite (first – black lines and last (max) loading shown – red lines). Left panel corresponds to submicron vaterite particles (average size 0.3 µm), right panel corresponds to particles of micron size (average size 3.6 µm).

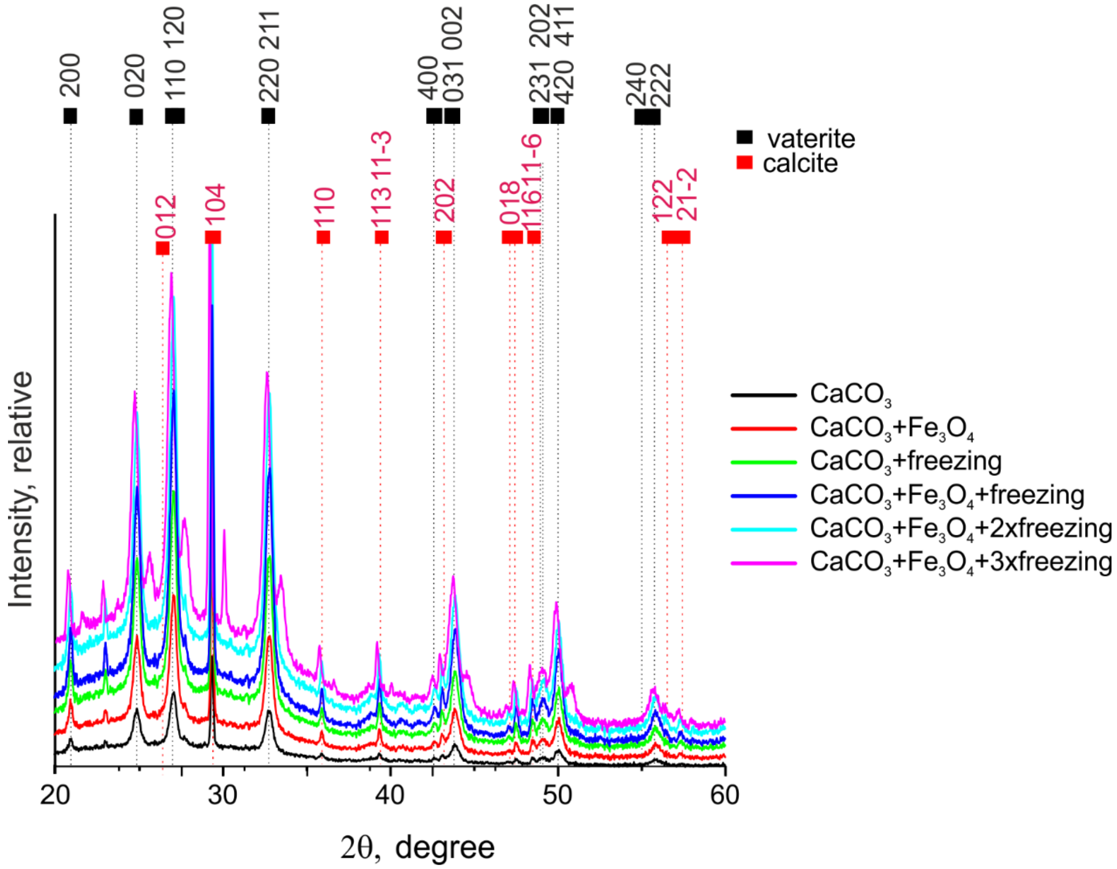


**Supplementary Figure S3.** Measurements for the XRD data obtained for the reference $CaCO_3$ and the composite $CaCO_3/Fe_3O_4$ crystals at room temperature (light blue curve), after 1 time (light green), 2 times (blue), and 3 times of freezing (green curve), and control XRD data of initial $CaCO_3$ particles at room temperature (black curve) and after freezing (red curve). Peaks indicated with black and red squares correspond to vaterite and calcite phases, respectively. The Miller indexes of each peak are highlighted on top.

**Supplementary Table S1.** Calcite to vaterite ratio in microparticles with and without magnetite measured by XRD after different number of freezing-thawing cycles.

| compound | freezing-thawing cycles | calcite/vaterite |
|---|---|---|
| **$CaCO_3$** | 0 | 1/6.4 |
| **$CaCO_3$+MNPs** | 0 | 1/7.8 |
| **$CaCO_3$** | 1 | 1/7.7 |
| **$CaCO_3$+MNPs** | 1 | 1/7.8 |
| **$CaCO_3$+MNPs** | 2 | 1/7.0 |
| **$CaCO_3$+MNPs** | 3 | 1/8.2 |

**Supplementary Table S2.** Adsorption efficiency of 10, 20 and 40 nm sized gold nanoparticles, where FF – filling factor is a ratio of surface area of cross section of adsorbed nanoparticles to the surface of vaterite particles evaluated using the equation

| number of loading | FIL | | | | | | Adsorption | | | | | |
|---|---|---|---|---|---|---|---|---|---|---|---|---|
| | 10 nm | | 20 nm | | 40 nm | | 10 nm | | 20 nm | | 40 nm | |
| | Δm, µg | FF | Δm, µg | FF | Δm, µg | FF | Δm, µg | FF | Δm, µg | FF | Δm, µg | FF |
| **1** | 390 | 3.2 | 660 | 2.8 | 700 | 0.7 | 130 | 1.1 | 580 | 2.3 | 210 | 0.27 |
| **2** | 285 | 2.3 | 650 | 2.7 | 620 | 0.7 | 100 | 0.7 | 220 | 1 | 250 | 0.27 |
| **3** | 300 | 2.4 | 600 | 2.5 | 700 | 0.7 | 80 | 0.6 | 20 | 0.06 | 270 | 0.27 |
| **total** | 980 | | 1920 | | 2010 | | 310 | | 820 | | 730 | |

The percentage of fill layer was calculated by means of the formula:

$$FF = \frac{N \cdot R_{AuNP}^2}{0.74 \cdot R_{CaCO_3}^2},$$

where is $N$- number of AuNPs, $R_{AuNP}$ – radius of AuNP, $R_{CaCO_3}$ – radius of vaterite particles.

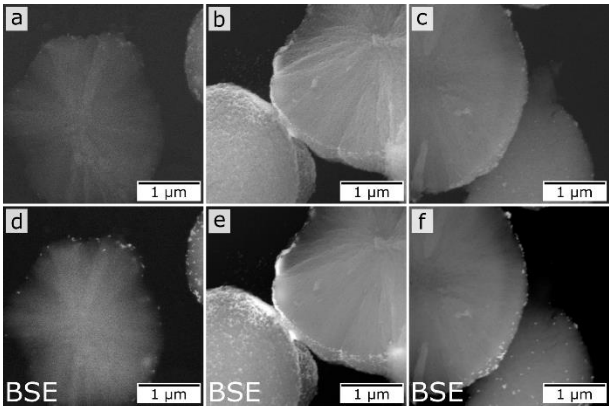


**Supplementary Figure S4.** SEM images of microparticles after freezing-induced loading of gold nanoparticles with a size of 10 (a, d), 20 (b, e) and 40 (c, f) nm. The d, e, and f are back-scattered electrons SEM images.

**Supplementary Equation S1.** Total surface area ratio of submicro- and microparticles at the same mass. This calculation considers only shape and size of particles and does not consider the porosity of particles.

$$\frac{S_{smp}}{S_{mp}} = \frac{\pi r_{mp}}{2ab}\left(b + \frac{a^2}{\sqrt{a^2-b^2}} arcsin\frac{\sqrt{a^2-b^2}}{a}\right),$$

where $S_{smp}$ – submicroparticles total surface, $S_{mp}$ – microparticles total surface, $r_{mp}$ – microparticle radius, $a$ and $b$ – semi-axes of submicroparticles (semi-axes of prolate spheroid).

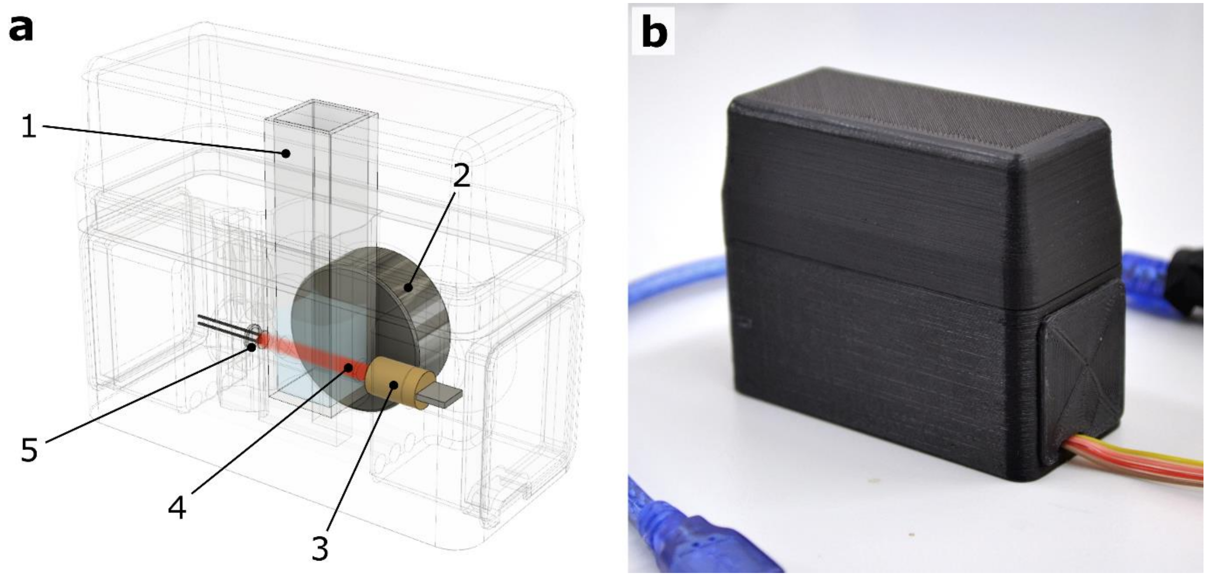


**Supplementary Figure S5.** Device for investigation of particle sedimentation rate. a – scheme of photometer. 1 – cuvette with particles suspension; 2 – permanent magnet; 3 – semiconductor laser; 4 – laser beam; 5 – phototransistor. b – photo of assembled device.

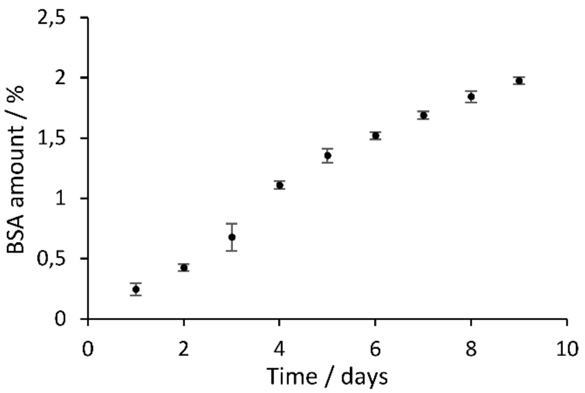


**Supplementary Figure S6.** Release of BSA-RITC from microcapsules during 9 days after microcapsule synthesis.
